# Coupled Evolution of Transcription and mRNA Degradation

**DOI:** 10.1371/journal.pbio.1001106

**Published:** 2011-07-19

**Authors:** Mally Dori-Bachash, Efrat Shema, Itay Tirosh

**Affiliations:** 1 Department of Molecular Genetics, Weizmann Institute of Science, Rehovot, Israel; 2 Department of Molecular Cell Biology, Weizmann Institute of Science, Rehovot, Israel; University College London, United Kingdom

## Abstract

mRNA levels are determined by the balance between transcription and mRNA degradation, and while transcription has been extensively studied, very little is known regarding the regulation of mRNA degradation and its coordination with transcription. Here we examine the evolution of mRNA degradation rates between two closely related yeast species. Surprisingly, we find that around half of the evolutionary changes in mRNA degradation were coupled to transcriptional changes that exert opposite effects on mRNA levels. Analysis of mRNA degradation rates in an interspecific hybrid further suggests that opposite evolutionary changes in transcription and in mRNA degradation are mechanistically coupled and were generated by the same individual mutations. Coupled changes are associated with divergence of two complexes that were previously implicated both in transcription and in mRNA degradation (Rpb4/7 and Ccr4-Not), as well as with sequence divergence of transcription factor binding motifs. These results suggest that an opposite coupling between the regulation of transcription and that of mRNA degradation has shaped the evolution of gene regulation in yeast.

## Author Summary

The regulation of mRNA levels in the cell is important to ensure, for instance, timely cellular responses to changes in the environment. mRNA transcription and mRNA degradation directly affect mRNA levels and it would make sense to have a system in place that would coordinate these opposing processes. Previous studies suggested that regulation of transcription in the nucleus may be linked to regulation of mRNA degradation in the cytoplasm, yet the details of this connection are poorly understood. In this study, we took an evolutionary approach to address this question by comparing both transcription and mRNA degradation between two yeast species. We found that evolution of these distinct processes is coordinated, as genes that diverged in mRNA degradation tend to also diverge in transcription. Interestingly, the coordination is counterproductive, as increased transcription is linked to increased mRNA degradation. We analyzed a hybrid between the two yeast species to classify evolutionary differences according to the type of underlying mutation (*cis* or *trans*). This analysis indicated that coordinated changes in transcription and mRNA degradation are likely to be driven by the same individual mutations, and thus directly coupled. Finally, we suggest several mechanisms that may mediate this coupling, including complexes which are involved in both processes (Rpb4/7 and Ccr4-Not) and promoter regulatory regions. These results suggest that a direct coupling between the regulation of transcription and mRNA degradation is a common phenomenon employed by approximately 10% of the genes in yeast.

## Introduction

Work on the regulation of mRNA levels has traditionally focused on transcription, although mRNA levels reflect the balance between transcription and mRNA degradation. Recent studies have shown that regulation of mRNA degradation also has a central role in control of gene expression, and in certain systems might be as important as transcription regulation [1]–[10], underscoring the importance of systematically studying the patterns of mRNA degradation and their regulation. While the basic machinery of mRNA degradation is well established [2],[11], very little is known regarding gene-specific and condition-specific regulation by RNA-binding proteins (RBPs), which bind to subsets of mRNAs and coordinate their post-transcriptional regulation [12],[13]. Notably, hundreds of RBPs are predicted in each eukaryotic genome, yet the subsets of bound mRNAs and the impact on mRNA degradation are known only for a selected few [14]–[18].

Although both transcription and mRNA degradation individually contribute to the regulation of mRNA levels, they are ultimately integrated to form a coherent regulatory system, and several studies provided evidence for crosstalk between the regulation of transcription and mRNA degradation. First, two conserved and general regulatory complexes, the Rpb4/7 dimmer, which is composed of two subunits of RNA polymerase II [19], and the Ccr4-Not complex [20],[21], have been shown to control both transcription and mRNA degradation and thus may serve to coordinate their regulation. Second, recent work in the fission yeast has described a feed-forward loop whereby a transcription factor activates a regulator of mRNA degradation and both factors jointly control the expression of a common subset of genes [22]. Such interplay between factors that control transcription and mRNA degradation might in fact be a common property of regulatory networks [23]. Third, several studies examined the response of *S. cerevisiae* to environmental perturbations and found coordinated changes in mRNA degradation and transcription [5],[6],[8],[9],[24]. For example, Shalem et al. [24] found that transcriptional regulation is coordinated with changes in mRNA stability and that the mode of this coordination is condition-dependent, such that induced genes are stabilized in one condition (during DNA damage) and destabilized in another (during oxidative stress).

Taken together, these observations suggest that transcription and mRNA degradation are often coordinated. However, this coordination remains poorly understood, raising several important questions. What is the scope of this coordination? What mechanisms underlie this coordination and are they directly or indirectly influencing both processes? What is the mode of coordination—is transcriptional induction mostly coordinated with decreased degradation, increased degradation, or both? What is the functional significance of such coordination?

To address these questions, we set out to examine the coordination between transcription and mRNA degradation from an evolutionary perspective, by comparing two closely related yeast species, *S. cerevisiae* and *S. paradoxus*. These species diverged from a common ancestor ∼5–10 million years ago and maintained similar physiology and genomic sequences (∼90% identity), yet as we have shown previously [25], most of their orthologous genes have diverged in mRNA levels. Comparing the mRNA degradation rates of these species, we find significant differences at ∼11% of the orthologs. Remarkably, around half of these evolutionary differences in mRNA degradation are coupled to evolutionary differences in transcription, indicating a widespread coordination. This coordination involves almost exclusively opposite effects of transcription and degradation such that transcriptional induction is coupled to increased mRNA degradation. Furthermore, classification of transcription and degradation changes into *cis* and *trans*, by allele-specific analysis of the interspecific hybrid, suggests a direct mechanistic coupling whereby individual mutations influence both transcription and mRNA degradation. These mutations seem to involve Rpb4/7, Ccr4-Not, as well as additional unknown factors.

## Results

### Genome-Wide mRNA Degradation Rates in Two Closely Related Yeast Species

**Figure 1:**
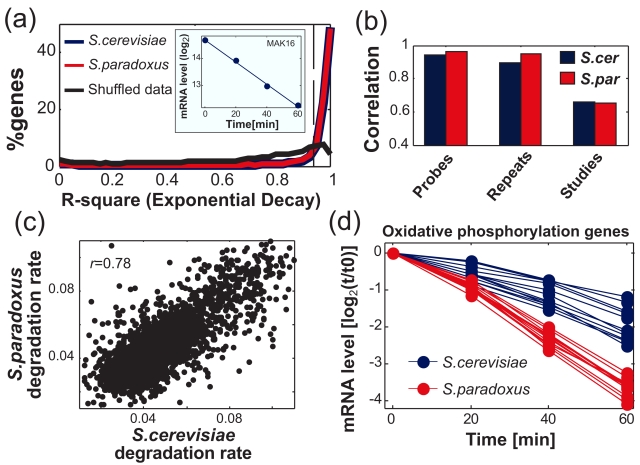
Large-scale analysis of mRNA degradation rates in two yeast species. (a) *R* ^2^ values (goodness-of-fit) for a linear-fit to the log_2_ mRNA levels at the four time points (see inset for example of a single gene). As control, we performed the same analysis to 10,000 shuffled profiles in which each time-point is taken from a different gene (randomly selected), thus retaining the overall degradation of mRNA levels but shuffling the gene-specific degradation rates. 78% of the real profiles (compared with 18% of the shuffled profiles) obtained an *R* ^2^ value above 0.94 and were included in all further analyses. (b) Correlation of *S. cerevisiae* (blue) and *S. paradoxus* (red) mRNA degradation rates: (i) between different probes for the same genes (note that different probes typically have different hybridization intensities, yet the mRNA degradation rates are highly reproducible, see Materials and Methods), (ii) between biological repeat experiments, and (iii) between this work and a previous work that used a temperature-sensitive mutation in RNA polymerase II to block transcription. Note that although this previous work analyzed only *S. cerevisiae*, it has high correlations with our data for the two species. (c) Scatter-plot of mRNA degradation rates in *S. cerevisiae* and *S. paradoxus*, which have a genome-wide correlation of 0.78. (d) Patterns of mRNA degradation for the 12 oxidative phosphorylation genes included in the analysis in *S. cerevisiae* (blue) and *S. paradoxus* (red).

To compare the mRNA degradation rates of the two species, we monitored mRNA levels following transcriptional arrest using 1,10-Phenantroline [7],[26]. mRNA levels were measured at 0, 20, 40, and 60 min after addition of the drug using a two-species microarray [25]. As expected, the profiles of most genes were well approximated by an exponential decay, which is reflected by a linear decrease of the log_2_ mRNA levels with time (Figure 1a). Degradation rates were estimated as the slope of the linear fit for 78% of the genes that had an *R* ^2^ value (goodness-of-fit) above 0.94, while genes with lower *R* ^2^ were excluded from further analysis. The calculated mRNA degradation rates of *S. cerevisiae* genes were highly reproducible among two biological repeats and between probes that were designed for different positions of the same genes, and were consistent with previous measurements of mRNA degradation that utilized a PolII mutant strain to block transcription (Figure 1b) [24].

Degradation rates were largely conserved among the two yeast species, with a genome-wide correlation of 0.78 (Figure 1c), yet we identified considerable differences at ∼11% of the orthologs, in which the difference was both statistically significant (*p*<0.05) and above 1.4-fold (i.e., the higher degradation rate exceeded the lower degradation rate by at least 40%, see Figure S1 for results with other thresholds). Differential mRNA degradation rates of six genes were validated by real-time PCR (Figure S2). These results indicate that, even among such closely related species, considerable differences in mRNA degradation rates are common, although much less common than differences in mRNA levels, which were observed for approximately half of the genes in this and in previous work (Figure S1) [25]. Differential degradation was observed for genes with various functions but was particularly enriched among respiration-related genes. Notably, degradation rates of these genes were consistently higher in *S. paradoxus* than in *S. cerevisiae*, as shown in Figure 1d for the 12 oxidative phosphorylation genes included in our analysis.

### Coupled Evolutionary Changes in Transcription and mRNA Degradation

**Figure 2:**
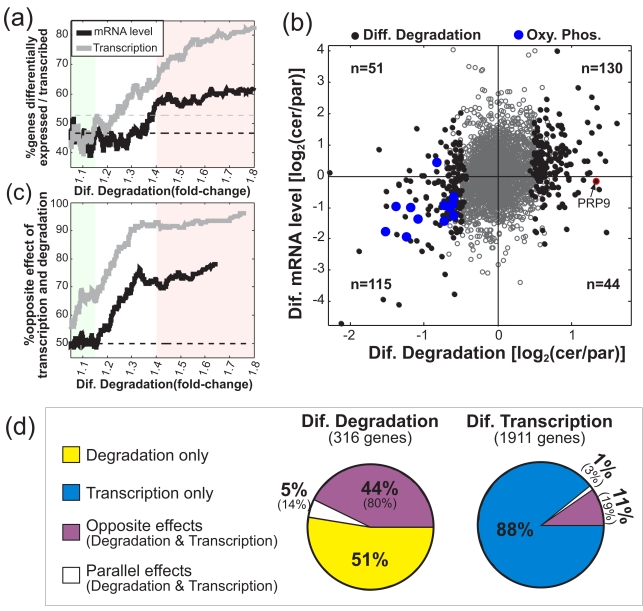
Coupled evolution of transcription and mRNA degradation. (a) Sliding window analysis (windows of 200 genes) for the percentage of inter-species differentially expressed genes (above 1.5-fold), using either mRNA levels (black) or estimated transcription rates (gray), as a function of the fold-change of inter-species differences in mRNA degradation rates. Dashed lines indicate the genome-wide percentage of differential mRNA levels (black) or transcription rates (gray). The green section includes small differences in mRNA degradation which may reflect technical variations, while the red section includes larger and biologically meaningful differences in mRNA degradation. (b) Scatter-plot of differential mRNA degradation rates versus differential mRNA levels for genes with (full circles) or without (empty circles) significant difference in mRNA degradation, and for oxidative phosphorylation genes (blue). The number of genes with significant difference in mRNA degradation is shown for each quarter, demonstrating an enrichment of genes with opposite effects of mRNA degradation and mRNA levels (genes with higher degradation in *S. cerevisiae* also tend to have higher mRNA levels, as the upper-right quarter has more genes than the lower-right quarter). (c) Sliding window analysis (windows of 200 genes) for the percentage of genes with opposite effects of transcription and degradation among those with differential mRNA degradation rate and either differential mRNA levels (black) or differential transcription rate (gray), as a function of the fold-change of inter-species differences in mRNA degradation rates. Dashed line indicates 50% opposite effects, as would be expected by chance if differential expression and differential degradation are independent. Green and red sections are as in (a). (d) Pie charts for the different combinations of differences in transcription and mRNA degradation, among the genes with differential mRNA degradation (right) and the genes with differential transcription (left). The analysis was performed with conservative estimates of coupling (only those with coupling as defined both by analyses of mRNA levels and by analysis of estimated transcription rates), while the percentages in parentheses show the results of a more relaxed analysis, in which either mRNA levels or transcription rates were sufficient to define coupling.

We next turned to systematically compare the changes in mRNA degradation rates to the changes in mRNA levels, as measured here in the zero time-point (before transcription arrest), or in a previous work [25]. Sorting the genes by the degree of inter-species differential degradation rate, we observed that differential degradation is associated with inter-species differential mRNA level (Figure 2a). This might seem expected, as mRNA levels are partially determined by mRNA degradation. Surprisingly, however, the direction of differences in mRNA levels is opposite to that expected purely from the difference in mRNA degradation: genes with higher mRNA degradation rate in one of the species tend to have *higher* mRNA levels in that species, although the increased degradation would be expected to decrease their mRNA levels (Figure 2b). This indicates that apart from the differences in degradation rates, there are also differences in the transcription rates of these genes that exert opposite effects on mRNA levels. For example, oxidative phosphorylation genes have significantly faster mRNA degradation in *S. paradoxus* than in *S. cerevisiae*, yet 11 out of 12 of these genes in fact have significantly higher mRNA level in *S. paradoxus* than in *S. cerevisiae* (Figure 2b, blue dots). Strikingly, in close to 80% of the genes with differential mRNA level and differential degradation, the difference in mRNA level is opposite to that expected from the difference in mRNA degradation, thus implying opposing effects of transcription and degradation (red section in Figure 2c).

Technical biases do not seem to have a significant effect on the observed coupling. First, the coupling is observed for large differences in mRNA degradation (red section), but not for genes with very small changes in degradation, which are more dependent on technical variations (green section in Figure 2c). Second, we used different datasets to compute mRNA levels and mRNA degradation, thus avoiding potential artifacts that might generate the observed coupling. Third, our microarray contains different probes for the same genes with widely different hybridization intensities (which serve to calculate mRNA levels), but these differences do not affect the estimation of mRNA degradation rates (see Materials and Methods). Fourth, the observed coupling cannot be accounted by microarray artifacts or residual transcription (see Methods and Figure S3).

Notably, the above analysis in fact underestimates the scope of the coupling between transcription and mRNA degradation, since mRNA levels are used instead of transcription rates. For example, some genes displayed a difference in mRNA degradation rates but no significant difference in mRNA levels (e.g., PRP9, see Figure 2b). This again implies an opposite difference in transcription that compensates for the difference in mRNA degradation (thus resulting in similar mRNA levels in the two species), yet these genes were not considered in our previous analysis. To account for this effect we can estimate the transcription rates of the two species by integrating the measures of mRNA levels and degradation (see Materials and Methods). This analysis indeed increases the proportion of coupled genes (gray curves in Figure 2a,c), although calculated transcription rates should be taken with caution and may artificially overestimate the coupling (see Materials and Methods). We thus predict the true scope of opposite coupling to be within the range indicated by analysis of mRNA levels and that of estimated transcription rates (e.g., among genes that differ both in transcription and in mRNA degradation ∼80%–90% have opposite effects; see Figure 2c). Nevertheless, in subsequent analyses we took a conservative approach and considered coupling only among those genes identified by both mRNA levels and estimated transcription rates.

Taken together, a large fraction of the evolutionary changes in mRNA degradation were coupled to opposite evolutionary changes in transcription (44%–80%, as derived from our conservative and relaxed analyses, respectively; see Figure 2d). Note, however, that this coupling constitutes only 10%–20% of the evolutionary changes in transcription (Figure 2d), as transcriptional changes were much more frequent and typically independent of those in mRNA degradation; this might explain why previous studies failed to notice such coupling.

### Hybrid Analysis Supports a Mechanistic Coordination between Transcription and mRNA Degradation

Transcription and mRNA degradation are controlled by different mechanisms and are thus expected to diverge through a separate set of mutations. However, the strong coupling that we observe suggests the intriguing possibility that individual mutations may influence both transcription and mRNA degradation, generating opposing effects on mRNA levels. Although we cannot identify the effect of individual mutations, this possibility can be examined by differentiating between the contributions of *cis*- and *trans*-mutations to evolutionary changes in mRNA degradation and transcription. *Cis*-mutations occur within the affected gene or in its flanking regulatory sequences (e.g., promoter or 3′-UTR motifs), while *trans*-mutations occur in other loci and indirectly influence the affected gene through the activity of another protein (e.g., RNA-binding protein). Importantly, the genome-wide contributions of *cis*- and *trans*-mutations can be uncovered by analysis of inter-species hybrids: *cis*-mutations discriminate between two hybrid alleles that reflect orthologs from the two species, while *trans*-mutations do not discriminate between the two hybrid alleles, as the alleles are in the same nucleus and thus exposed to the same set of *trans*-regulators. This approach has previously been used to assess the contribution of *cis*- and *trans*-mutations to total mRNA levels [25],[27]–[29] and recently also to nucleosome positioning [30], while here we extend it to study mRNA degradation rates.

**Figure 3:**
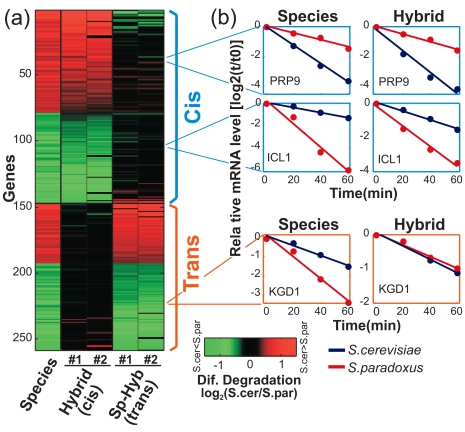
Cis and trans divergence of mRNA degradation. Classification of inter-species differences in mRNA degradation rates into *cis* and *trans* based on the extent of differences observed between the two hybrid alleles (see Materials and Methods). (a) Heatmap of the differences in mRNA degradation rates, log_2_(*S. cer*/*S. par*), between the two species (left column), between the corresponding hybrid alleles (middle columns, reflecting only the *cis* component), and the subtraction of the species and hybrid differences (right columns, reflecting only the *trans* component). (b) mRNA degradation profiles of the two species (left) and the corresponding hybrid alleles (right) are shown for two examples of *cis*-differences (top) and one example of *trans*-difference (bottom).

We measured allele-specific mRNA degradation rates for the hybrid of *S. cerevisiae* and *S. paradoxus*, with two biological repeats and using the same method as described above for the two species. For each gene whose mRNA degradation rate differs between the species, we examined whether this difference is maintained (*cis*) or abolished (*trans*) between the corresponding two hybrid alleles. This analysis indicated that ∼60% of the differences in mRNA degradation reflect primarily *cis*-mutations, while ∼40% reflect *trans*-mutations (Figure 3). Six *cis*-differences were further validated by real-time PCR of the hybrid alleles (Figure S2).

**Figure 4:**
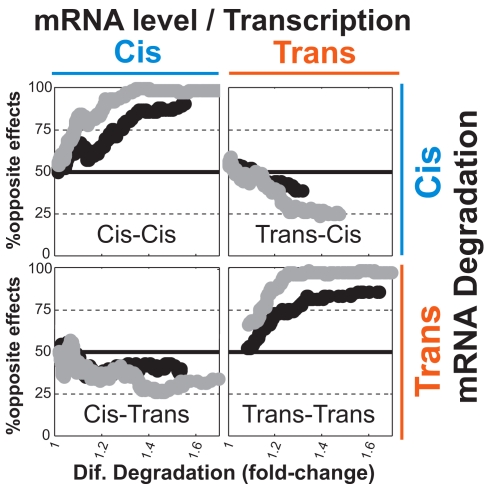
Enrichment of opposite effects only for *cis*-*cis* and *trans*-*trans* combinations supports a mechanistic coupling. Inter-species differences in mRNA levels (or estimated transcription rates) and mRNA degradation were divided into the contribution of *cis*- and *trans*-mutations based on the hybrid data. The enrichment of opposite transcription and degradation effects was examined for each of the four combinations of *cis* and *trans*, by a sliding window analysis of the percentage of opposite effects as a function of the fold-changes in mRNA degradation.

If coupled changes in transcription and degradation are due to independent mutations, then each change can be either in *cis* or in *trans*, and thus the coupling should be observed for all combination of *cis*- and *trans*-effects; for example, *cis*-effects in mRNA degradation should be coupled both to *cis*-effects in transcription (*cis*-*cis* combination) and to *trans*-effects in transcription (*cis*-*trans* combination). However, if transcription and degradation changes are mechanistically coupled and the observed opposite effects are generated by the same individual mutations, then these coupled changes would be generated by a single effect, either in *cis* (*cis*-*cis* combination) or in *trans* (*trans-trans* combination), but not by *cis*-*trans* or *trans*-*cis* combinations. Consistent with this, a strong coupling is observed only for *cis*-*cis* and *trans*-*trans* combinations but not for *cis*-*trans* or *trans*-*cis* combinations (Figure 4).

### *Trans*-Factors Associated with Coupling of Transcription and mRNA Degradation

**Figure 5:**
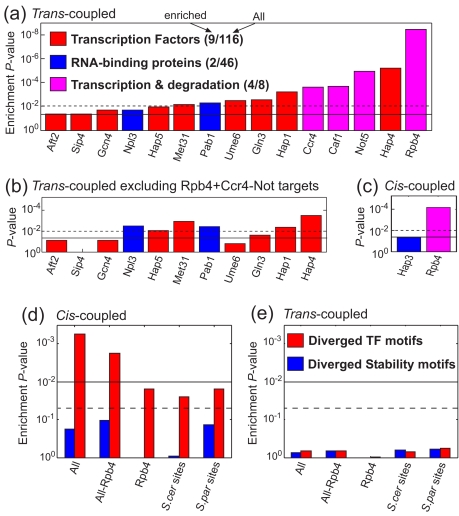
Coupling is associated with divergence of Rpb4/7, Ccr4-Not, and TF motifs. (a) Target-sets of various TFs [31], RNA-binding proteins [14], and two complexes implicated in both transcription and degradation (Rbp4/7 [32] and Ccr4-Not [33]) were examined for enrichment with *trans*-coupled genes. 15 and 10 datasets had significant enrichment below a *p* value of 0.05 (full line) and 0.01 (dashed line), respectively, and these are shown in order of statistical significance. The total numbers of analyzed datasets and those with significant enrichments are shown in parentheses. (b) Same as in (a) after excluding targets of Rpb4, Ccr4, and Not5. (c) Same as in (a) for enrichment with *cis*-coupled genes. (d) Diverged TF binding [31] (red) or mRNA stability [15] (blue) motifs, which are intact only in *S. cerevisiae* (*S. cer* sites) or only in *S. paradoxus* (*S. par* sites), were identified by sequence analysis. The enrichment of diverged motifs (for all TFs combined or all stability motifs combined) was examined among all *cis*-coupled genes (All), *cis*-coupled genes predicted to be targets (Rpb4) or non-targets (All-Rpb4) of Rpb4, and for *cis*-coupled *S. cer* sites or *cis*-coupled *S. par* sites. (e) Same as (d) for enrichment with *trans*-coupled genes.

Coupling between *trans*-changes in mRNA degradation and *trans*-changes in transcription (*trans*-coupling) suggests that divergence of upstream regulator(s) has influenced both processes. We thus searched for enrichment of 85 high-confidence *trans*-coupled genes with targets of 116 transcription factors (TFs) [31], 46 RNA-binding proteins (RBPs) [14],[16], Rpb4/7 [32], and Ccr4-Not [33]. Fifteen of the 173 target gene-sets were enriched (*p*<0.05) among the *trans*-coupled genes compared to uncoupled genes (Figure 5a). Notably, these include an Rpb4/7 dataset (Rpb4 [32]) and three datasets of Ccr4-Not (Ccr4, Not5, Caf1 [33]), which were among the five most enriched datasets. Furthermore, while the combined target gene-sets of Rpb4/7 and Ccr4-Not include only 12% of all genes examined here and 18% of the genes with uncoupled transcriptional changes, they include 41% of the *trans*-coupled genes (*p* = 2×10^−7^). Thus, our results are consistent with previous studies showing that these two complexes influence both transcription and mRNA degradation.

Target gene-sets of nine TFs and two RBPs were also enriched with *trans*-coupled genes (Figure 5a). However, excluding the targets of Rpb4/7 and Ccr4-Not completely abolished the enrichment of four of these TFs (Figure 5b), suggesting that their enrichment was due to high overlap with targets of Rpb4/7 and Ccr4-Not and may not reflect the function of these TFs. The remaining enriched gene-sets included targets of three TFs involved in respiration (Hap1, Hap4, and Hap5), two TFs involved in amino-acid biosynthesis (Gln3, Met31), the poly(A) binding protein (Pab1), and the SR-like protein Npl3. Interestingly, both Pab1 [34] and Npl3 [35] are known to shuttle between the nucleus and the cytoplasm, Npl3 was previously implicated in regulation of transcription [36] and translation [37], and Pab1 was previously implicated in regulation of mRNA degradation [38]. These results suggest that, in addition to Rbp4/7 and Ccr4-Not, coordination between transcription and mRNA degradation may also involve Pab1 and Npl3.

The enrichment of *trans*-coupled genes among targets of specific regulators suggests not only that these regulators control both transcription and mRNA degradation, but also that the activity of these regulators diverged among the two species. Consistent with this possibility, the expression level of Rpb4 is ∼3-fold higher in *S. paradoxus* than in *S. cerevisiae*, while the expression of other RNA Pol II subunits is much more conserved (Figure S4). Increased activity of Rpb4/7 in *S. paradoxus* would be expected to increase both transcription and mRNA degradation in *S. paradoxus* (compared to *S. cerevisiae*), and indeed we find that targets of Rpb4/7 are highly enriched among coupled *trans*-effects with higher *S. paradoxus* transcription and degradation but not among those with higher *S. cerevisiae* transcription and degradation (Figure S4).

### *Cis*-Elements Associated with Coupling of Transcription and mRNA Degradation

Coupling between *cis*-changes in mRNA degradation and *cis*-changes in transcription (*cis*-coupling) suggests that mutations in a gene's promoter, coding-region, terminator or untranslated regions influenced both processes. This may reflect mutations that affect the recruitment of specific proteins to the loci of that gene, which then influence both transcription in the nucleus and degradation of the resulting mRNA following its export to the cytoplasm. To examine this possibility, we first searched for enrichment of 92 high-confidence *cis*-coupled genes with targets of the various regulators, as described above for the *trans*-coupled genes. Only one of the 170 datasets was enriched among the *cis*-coupled genes with a *p* value below 0.01 (Figure 5c). This dataset included genes upregulated upon deletion of Rpb4 and was significantly enriched with *cis*-coupling (*p* = 7×10^−5^), suggesting that *cis*-mutations may have influenced the recruitment of Rpb4/7 to many genes. At a *p* value of 0.05, only one additional target gene-set was enriched (Hap3), while ∼9 sets would be expected by pure chance (0.05×173).

Despite the significant enrichment of Rpb4/7 targets, these include only 13% of the *cis*-coupled genes, suggesting the existence of other mechanisms for *cis*-coupling. We next examined the sequence divergence between *S. cerevisiae* and *S. paradoxus* at various predicted and known *cis*-regulatory elements. Analysis of diverged 3′-UTR sequences that were predicted to influence mRNA stability [15] or to be bound by RNA-binding proteins (RBPs) [14],[16] did not identify a significant association with *cis*-coupled genes (Figure 5d). In contrast, diverged transcription factor (TF) binding sites [39] were significantly enriched at *cis*-coupled genes, compared to uncoupled genes that diverged only in transcription (Figure 5d, *p*<10^−3^). This enrichment was found both for known *S. cerevisiae* TF binding sites [31] that are not conserved in *S. paradoxus* and for predicted *S. paradoxus* TF binding sites that are not conserved in *S. cerevisiae* (Figure 5d). Notably, diverged TF binding sites were enriched at *cis*-coupled target genes of Rpb4/7, suggesting that these mutations may have influenced the recruitment of Rpb4/7, but also at *cis*-coupled genes not targeted by Rpb4/7, implying that the effect of these mutations on transcription and mRNA degradation is also mediated by additional mechanisms. This analysis of diverged binding sites included various TFs and we could not detect any TF with specific overrepresentation. As expected, diverged TF binding sites were not enriched among *trans*-coupled genes (Figure 5e), further supporting their direct association with *cis*-coupling.

## Discussion

Our systematic comparison of mRNA degradation among two yeast species demonstrated the following: (i) Degradation rates differ among ∼11% of the orthologs, compared to ∼50% that differ in transcription or in mRNA levels. (ii) Differences in mRNA degradation are often coupled to opposite differences in transcription, and this coupling constitutes around half of the changes in mRNA degradation but only ∼10% of the changes in transcription. (iii) Coupled changes in transcription and degradation are generated by the same type of mutations (*cis* or *trans*) suggesting a mechanistic coupling. (iv) *Trans*-coupling is associated with regulators that are known (Rpb4/7 and Ccr4-Not) to control both transcription and mRNA degradation, while *cis*-coupling may be associated with diverged TF motifs.

### *Trans*-Coupling through Parallel Regulation of Transcription and mRNA Degradation

**Figure 6:**
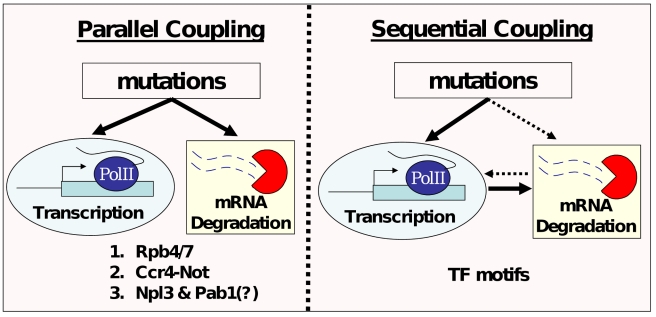
Two models of mechanistic coupling whereby individual mutations affect both transcription and mRNA degradation. The first model (Parallel coupling, left) assumes mutations in a single *trans*-factor that influences both processes and is consistent with the enrichment of *trans*-coupled genes with targets of Rpb4, Ccr4-Not, Pab1, and Npl3. The second model (Sequential coupling, right) assumes mutations that exert transcriptional effects (either in *cis* or in *trans*) and that these transcriptional effects then induce changes in mRNA degradation, for example, through a shuttling mechanism whereby Rpb4/7 (or other transcription-related molecules) binds to the mRNA co-transcriptionally and transports with it to the cytoplasm. This model is consistent with the enrichment of diverged TF motifs among cis-coupled genes. An opposite sequential coupling is also possible (dashed arrows), whereby mutations affect mRNA degradation and this effect then induces transcriptional changes, yet we do not find evidence to support it.

The association of *trans*-coupled genes with Rpb4/7 and Ccr4-Not suggests that altered activity of these complexes influenced, in parallel, both transcription and mRNA degradation of target genes. This possibility of parallel coupling (see Figure 6), whereby an upstream regulator controls multiple regulatory steps and may coordinate them, is consistent with known functions of Rpb4/7 and Ccr4-Not and, more generally, with the notion that RBPs often coordinate multiple steps in the regulation of their target genes [13]. *Trans*-coupling is also associated with two other RBPs known to shuttle between the nucleus and the cytoplasm (Pab1 and Npl3), suggesting that these may also serve as coordinators of transcription and mRNA degradation, and possibly of additional steps.

Notably, divergence of individual *trans*-regulators can cause similar evolutionary changes across many co-regulated target genes. Indeed, *trans*-coupling includes a set of respiration-related genes, all with higher transcription and mRNA degradation rates in *S. paradoxus* than in *S. cerevisiae*, likely reflecting a module that coherently diverged through one or few *trans*-mutations. While this module is known to be transcriptionally co-regulated, these results suggest that it is also post-transcriptionally co-regulated, thus representing an “RNA regulon” [13]. Divergence of this module may have been part of the domestication of *S. cerevisiae* and an associated optimization of anaerobic fermentation [40]. Notably, although high-confidence *trans*-coupled genes are highly enriched with the respiration module (*p* = 10^−10^), this enrichment accounts only for a quarter (21/85) of these genes, suggesting that additional RNA regulons might have evolved by parallel (and opposite) changes in their transcription and mRNA degradation.

### *Cis*-Coupling May Involve Sequential Regulation of Transcription and mRNA Degradation

While *trans*-regulators may affect transcription and mRNA degradation in parallel, *cis*-acting sequences are likely to be more specific to one of these processes, for example, by mediating the binding of TFs to promoters or that of RBPs to mRNAs. We thus propose that *cis*-coupling may work by sequential coupling (Figure 6), whereby mutated *cis*-acting elements affect one process (transcription or degradation) and this in turn signals to the other process, thereby causing an additional effect. The enrichment of *cis*-coupling with diverged TF motifs, but not RBP (i.e., stability) motifs, suggests a mode of sequential coupling that is directed from transcription to mRNA degradation. This possibility is consistent with a shuttling mechanism, as previously proposed for Rpb4/7 [19], whereby transcription-related molecules bind to the transcribed mRNA and are exported with it to the cytoplasm where they influence its degradation. Rpb4/7 targets are indeed enriched among *cis*-coupled genes, but this accounts only for a small proportion of *cis*-coupling, suggesting the existence of additional factors for sequential coupling by a similar shuttling mechanism or by other mechanisms.

Alternatively, the enrichment of TF motifs, but not stability motifs, may reflect the bias in current knowledge, as fewer motifs are known for RNA-binding proteins and these may rely more heavily on structural properties. Sequential coupling may thus initiate by binding of RBPs to yet unknown motifs and regulate mRNA degradation, followed by signaling back to the nucleus that influences transcription of that gene or perhaps of a set of genes. This possibility is consistent with the notion that RBPs are highly inter-connected and coordinate multiple regulatory events [13]. However, the observation that coupling typically involved larger changes in transcription than in mRNA degradation appears to support a transcription-to-degradation directionality. Interestingly, both of these models make the intriguing and testable prediction that experimental manipulation of individual *cis*-regulatory elements would affect both transcription and mRNA degradation of the associated genes.

### The Scope and Mode of Coupling between Transcription and mRNA Degradation

The results presented here reflect the specific evolutionary divergence of two yeast species and hence might not be sufficient to infer general conclusions regarding the scope and mode of coupling. For example, few *trans*-mutations may have driven the evolution of many target genes (e.g., respiration module) and by that bias our results. Importantly, however, *cis*-coupled genes are each affected by distinct sets of mutations; the only exception is of neighboring genes which may diverge through the same mutations in *cis*, but these encompass only up to 5% of the observed *cis*-coupled genes. Therefore, our results imply ∼140 independent cases in which *cis*-acting mutations affected both transcription and mRNA degradation, generating opposite effects on mRNA levels (Figure 2d). At the same time, ∼1,700 genes diverged by *cis*-acting mutations only in transcription, and ∼160 genes diverged by *cis*-acting mutations only in mRNA degradation (Figure 2d). These results demonstrate that coupling is not a global phenomenon, as it does not affect the majority of genes, nor is it a rare event.

It is tempting to further speculate that *cis*-divergence is not strongly biased towards certain mechanisms and thus that observed patterns of *cis*-divergence may provide a rough estimate for the frequencies of possible mutational outcomes and regulatory mechanisms. Accordingly, we would predict that (i) transcriptional regulation is much more prevalent than regulation of mRNA degradation, although the exact proportion is difficult to quantify as differential mRNA degradation is more difficult to identify than differential transcription; (ii) coupling constitutes approximately 10% of the regulation of transcription but almost half of the regulation of mRNA degradation. (iii) Coupling occurs almost exclusively between opposite effects on mRNA levels (increased transcription is associated with increased mRNA degradation and vice versa).

This last prediction is especially surprising given that previous studies have highlighted a coherent mode of coupling whereby changes in mRNA levels may be driven by both transcription and mRNA degradation acting in the same direction [5],[7],[8],[22],[41]. These views may be reconciled if one mode (coherent changes) reflects coordination of distinct pathways for transcription and mRNA degradation that have co-evolved to support certain responses to environmental perturbations, while the other mode (opposite changes) reflects a mechanistic coordination whereby the same pathway affects both processes. Since these closely related species differ in the regulation of approximately half of the genes, and these differences are small in magnitude (∼1.5-fold), we suspect that they primarily reflect neutral drift and as such they expose the mechanistic (opposite) coupling that is presumably “built in” to regulatory mechanisms, but does not reveal coherent coupling as these primarily evolved prior to the divergence of these species and may not be continuously evolving.

### Implications of Opposite Coupling between Transcription and mRNA Degradation

This proposed mode of opposite coupling appears counterintuitive and inefficient, as transcription and degradation effects would compensate one another. What then may be the benefits of such coupling? One possibility is that an opposite coupling may enable transient responses to environmental changes: upon stress conditions, cells cease to grow and mount an transcriptional response, but at the same time increase the degradation rates of upregulated genes, thereby facilitating their return to basal expression levels and normal growth [4],[24]. Such transient responses may have been particularly important for thriving in fluctuating environments, and coupling mechanisms may have thus become “built-in” components of gene regulation that are active also in the absence of stress and are thus exposed by genetic mutations.

Another plausible advantage of such coupling is that it may decrease the effect of genetic or environmental perturbations on mRNA abundance, as changes in one level of regulation would be compensated by another level. Such intrinsic “negative feedback” could increase the robustness of gene regulation and thus reduce cell-to-cell variability. Surprisingly, however, we observe the exact opposite: genes that display coupled evolution in our data or that are targets of coupling mechanisms (i.e., Rpb4/7 and Ccr4-Not) have a considerably higher cell-to-cell variability in protein abundance (expression noise [42]) than other genes (Figure S5). Notably, this effect is comparable in magnitude to other factors that were previously implicated in increasing noise (i.e., TATA-box [43] and promoter nucleosome occupancy [44]) and remains significant after controlling for these factors. This may indicate that coupling between transcription and mRNA degradation is further associated with additional regulatory effects. Given the recent demonstration that Rpb4/7 also influences translational regulation [45], and the interplay between mRNA degradation and translation [46]–[48], it is tempting to speculate that the coupling that we observed is further linked to translation bursts that give rise to high cell-to-cell variability [49].

## Materials and Methods

### Strains and Growth Conditions

To facilitate comparison to the diploid hybrid, we generated diploid homozygote yeast strains of the two species, thus avoiding both potential differences between haploids and diploids, and potential heterozygosity within normal diploid strains, which could confound inter-species comparisons. Diploid homozygote strains were generated from the haploid *S. cerevisiae* (BY4741) and *S. paradoxus* (CBS432) strains, by transient HO activation and selection for diploid strains. The hybrid strain was generated by mating the same parental haploids. These three diploid strains (*S. cerevisiae*, *S. paradoxus*, and hybrid) were grown to log-phase at rich media (YPD medium at 30°C).

### Microarray Design

Two to five different 60-mer probes were designed for most genes in each of the two species, and each probe was placed at two different positions (duplicates) on an Agilent custom (two-species) microarray. Probes were selected both by general criteria for probe selection (intermediate %GC, no self-hybridization or low complexity regions, distance from the gene 3′-end) and by preference for low sequence similarity between the two species in order to avoid cross-hybridization (all probes reflect genomic positions with lower than 90% sequence identity between the two species).

### RNA Preparation, Microarray Hybridization, and Scanning

*S. cerevisiae*, *S. paradoxus*, and their hybrid were subjected to 150 µg/ml of 1,10-phenanthroline at log-phase and sampled after 0, 20, 40, and 60 min. Total RNA was extracted using MasterPure Yeast RNA purification Kit (EPICENTRE), amplified with Agilent's Low RNA Input Amplification Kit and hybridized with Agilent's standard protocols and kits to the two-species microarrays. *S. cerevisiae* and *S. paradoxus* samples were pooled and hybridized together and the hybrid was hybridized separately, both with biological repeats. Arrays were scanned using Agilent microarray scanner and feature extraction software. Raw and processed microarray data are available at the GEO database (GSE28849).

### Global Scaling of Microarray Data

During the time-course, transcription is arrested and total mRNA levels are decreasing, but this decrease is masked by the experimental protocol, as equivalent amounts of total RNA are extracted from each sample. Previous studies that used a PolII mutant strain could circumvent this problem since mRNAs constitute only a minor fraction of the total RNAs in a yeast cell, and the transcription of other RNAs (by PolI and PolIII) was not inhibited [3],[24]. However, Phenanthroline appears to inhibit all three RNA polymerases to approximately the same extent and we did not detect a decrease in the relative levels of mRNA (unpublished data). We therefore decided to scale the entire data at each time point according to an overall exponential decay with half-life of 25 min, consistent with previous studies [3],[24]. Accordingly, log_2_ of the total (or average) abundance of all mRNAs should decrease linearly by 1 unit every 25 min, and thus decrease by 0.8 every 20 min (the interval between consecutive time-points). We thus scaled the data by centering the four consecutive time points (0, 20 min, 40 min, and 60 min) on 0, −0.8, −1.6, and −2.4, respectively.

### Analysis of mRNA Degradation Rates

For each probe, we averaged the hybridization intensities from the duplicate microarray spots, and fitted a linear slope to the log_2_-intensities. All probes with an *R* ^2^ value smaller than 0.94 were excluded from further analysis. For each gene, the absolute value of the median slope of all remaining probes was defined as its degradation rate.

Since the four time-points are evenly spaced (0, 20, 40, and 60 min) the difference between mRNA levels at consecutive time-points should be approximately constant and reflect the mRNA degradation rates. To identify differential degradation rates among orthologous probes, we thus performed a two-sample *t* test, comparing the three estimates of each probe (M_20_–M_0_, M_40_–M_20_, and M_60_–M_40_, where M_i_ is the mRNA level at time i) between the two species. Genes for which the median *p* value from the *t* tests of the different probes was below 0.05 were further examined. *p* values reflect both the degree of differential degradation and the consistency among the three estimates (even a negligible difference can be identified as significant if the three measures are highly similar within each species). We thus further examined the extent of differential degradation and retained only those genes in which the ratio between the faster and lower degradation rates (from the two species) is higher than 1.4.

### Analysis of Steady-State mRNA Levels

The first time-point reflects mRNA levels during exponential growth and before transcriptional arrest. It therefore reflects an approximate steady-state mRNA level. A potential caveat is that if the first time-point is used to measure both mRNA levels and mRNA degradation, then measurement errors could generate artificial coupling between mRNA levels and degradation. For example, if the first time point is increased due to technical noise, then estimates of both mRNA level and mRNA degradation would increase and result in apparent coupling. To avoid this problem, we used only one time-course to derive estimates of mRNA degradation rates and the first time-point of the second time-course to derive an estimate of mRNA level. As additional control, we used mRNA levels as measured in a previous work and obtained similar results (unpublished data) [25]. Differential expression was defined as above 1.5-fold difference between the species (or hybrid alleles).

### Analysis of Transcription Rates

For each gene, we assume that the production rate of mRNAs (transcription rate) is approximately equal to the overall degradation of mRNAs, and therefore given by the steady-state level of mRNAs multiplied by their constant degradation rate. Hence, TR = D×L, where TR, D, and L are the transcription rate, degradation rate, and mRNA level, respectively. The difference in transcription rates between *S. cerevisiae* and *S. paradoxus* can thus be estimated from the respective differences of degradation rates and mRNA levels: log(TR_cer_/TR_par_) = log(D_cer_/D_par_)+log(L_cer_/L_par_).

We note that this estimation may not be accurate as a result of possible violation of the steady-state assumption, spurious correlations with mRNA degradation due to the method of calculation, and the integration of nuclear and cytoplasmic mRNAs in our measurements. Our main conclusions do not require these estimates of transcription rates and can be inferred from direct comparison of inter-species differences in mRNA degradation to those in mRNA levels. However, since mRNA levels are inherently affected by mRNA degradation in a manner that is opposite to the observed coupling, such analysis would underestimate the scope of the coupling (as illustrated in Figure 2c by PRP9). We thus argue that analysis of mRNA levels underestimates the scope of the coupling, while analysis of estimated transcription rates may overestimate it and that the two analyses are complementary. Nevertheless, we defined coupled genes for further analysis based on consensus of mRNA levels and transcription rates analyses in order to avoid cases of spurious coupling.

### Potential Confounding Effects

Our experimental design may be susceptible to two confounding effects. First, the use of two-species microarrays, whereby the two species are co-hybridized to a single array that contains species-specific probes, may result in cross-hybridization such that mRNA from one species hybridizes to probes of the other species. Second, inhibition of transcription with 1,10-phenanthroline may not be enough to completely block transcription and residual transcription activity may hinder our calculation of mRNA degradation rates. However, as described below, both of these effects are likely to have only a minor influence on our results and, in particular, are not expected to cause the observed coupling between transcription and mRNA degradation.

#### Microarray artifacts

The effect of cross-hybridization is minimized by our microarray design, where probes were selected for genomic regions with relatively low sequence similarity between the two species. The species-specificity of our probes was previously demonstrated by comparative genomic hybridization [25], and is further demonstrated by the high frequency of genes in which we observe significant differential expression between the two species. Notably, the remaining cross-hybridization should slightly diminish the extent of observed differential mRNA levels and differential mRNA degradation and thus result in underestimation of species differences. However, this effect is not expected to cause opposite changes in transcription and degradation.

As additional control for microarray artifacts, we note that most genes are assayed by multiple probes. These probes target different sequences inside genes, and due to the protocol's bias to the 3′-end (as a result of polyT primers), the probes have large differences in hybridization intensities. This effect does not influence our ability to identify inter-species differential expression, since we always compare orthologous probes that target the same positions, but it does provide us with a control for the effect of hybridization intensities: for most genes we have multiple probes with large differences in hybridization intensities. Importantly, despite the variability in hybridization intensities, we find that changes over the time-course (i.e., the rates of mRNA degradation) are highly correlated among the different probes (e.g., see Figure 1b).

#### Residual transcription activity

A constant residual transcription activity would cause genes to have a variable degradation rate during the time-course (non-linear decrease in their log_2_ mRNA levels): as mRNA degrades and the level of mRNA decreases, the relative contribution of residual transcription on the total mRNA levels gradually increases, until a new steady-state is reached in which the degradation rate is balanced by the residual transcription. Thus, for genes with constant residual transcription, the observed rate of mRNA degradation should decrease with time, and at some point, but possibly after longer than 60 min, should reach zero (i.e., the pattern of mRNA levels will reach a plateau). Similarly, a transient transcriptional activity would also cause genes to have variable degradation rates during the time-course. For example, if genes are transiently upregulated in response to addition of the drug but this response ceases before the end of the time-course, then during the transcriptional response the apparent degradation rate would be lower than after it ceases. A similar effect would also be expected if mRNA degradation rates are changed during the time-course, for example if degradation factors are affected by the drug or by the stress that is associated with transcriptional arrest. Taken together, residual transcription and additional potential artifacts would lead to a variable degradation rate during the time course and thus a low *R* ^2^ value of the linear fit to log_2_ mRNA levels.

These effects are minimized in our analysis by the stringent criteria for inclusion of genes only if their profile of log_2_ mRNA levels has an extremely good linear fit (*R* ^2^>0.94). To further verify that this effect is not generating the observed association between transcription and mRNA degradation, we used even more stringent criteria for inclusion of genes in the analysis (e.g., increased the threshold up to *R* ^2^>0.995) and obtained similar results (Figure S3). In fact, as the criteria for inclusion of genes became more stringent, the percentage of opposite effects in transcription and mRNA degradation further increased, suggesting that residual transcription might actually lead to underestimation of the coupling.

### Classification to *cis*- and *trans*-Changes in mRNA Degradation Rates

Classification into *cis* and *trans* is based on whether the inter-species difference in mRNA degradation rates (*Δspecies*) is retained (*cis*) or abolished (*trans*) within the hybrid (*Δhybrid*), while intermediate cases are excluded from the analysis. *Cis* changes were defined as significant inter-species differences for which *Δhybrid* has the same sign as *Δspecies* and is larger than 1.2-fold for each of the two repeats, and the residuals (*Δhybrid–Δspecies*) are smaller than 1.3-fold. *Trans* changes were defined as significant inter-species differences for which *Δhybrid* has either a different sign than *Δspecies* or is smaller than 1.2-fold for each of the two repeats, and the residuals (*Δhybrid–Δspecies*) are larger than 1.3-fold. This definition is clearly threshold dependent, and other thresholds or criteria that we used led to similar proportions of *cis* and *trans* changes, typically with the percentage of *cis* differences between 50% and 75% (unpublished data).

High-confidence sets of *cis/trans*-coupled genes were defined as those with a significant *cis*/*trans* mRNA degradation difference above 1.5-fold and a *cis*/*trans* mRNA level difference above 1.5-fold (in the opposite direction to that expected by the degradation difference).

### Target-Sets of Various Regulators

Targets of 116 TFs were defined based on Chromatin Immuno-precipitation and sequence analysis, taken from MacIsaac et al. [31] (*p*<0.005 and no conservation criteria). Targets of RNA-binding proteins were defined based on RNA Immuno-precipitation, taken from Hogan et al. [14]. Targets of seven subunits of Ccr4-Not were defined as genes whose expression decreased by at least 2-fold upon deletion of the respective subunits in rich media [33]. Targets of Rpb4/7 were defined as genes whose expression decreased by at least 2-fold upon deletion of Rpb4 in rich media [32].

### Diverged TF Binding Sites

TF binding motifs were taken from MacIsaac et al. [31] (*p*<0.005 and no conservation criteria).

Diverged binding sites were defined as follows:

#### Diverged in *S. paradoxus*

Among the known TF binding events in *S. cerevisiae*, we searched for those in which (i) the bound *S. cerevisiae* promoter contains a match to the respective PSSM which exceeds a LOD score of 10 and is at least 75% of the maximal LOD score for that PSSM in the entire genome. (ii) This motif is mutated in the orthologous *S. paradoxus* promoter such that the LOD score drops by at least one unit, and no other motif for that TF with higher LOD score is found in that *S. paradoxus* promoter.

#### Diverged in *S. cerevisiae*

For each TF, we examined all the genes which are not bound by that TF in *S. cerevisiae* and required the following: (i) The *S. paradoxus* promoter contains a match to the respective PSSM which exceeds a LOD score of 12 and is at least 90% of the maximal LOD score for that PSSM in the entire genome. (ii) This motif is mutated in the orthologous *S. cerevisiae* promoter such that the LOD score drops by at least 1.5, and no other motif for that TF with higher LOD score is found in that *S. cerevisiae* promoter.

Note that since we only have binding data in *S. cerevisiae*, our definition of diverged motifs is not symmetrical. We have identified more diverged motifs in *S. cerevisiae*, but these are of lower confidence as the binding in *S. paradoxus* is supported only based on the presence of the motif, while the binding in *S. cerevisiae* (for motifs that diverged in *S. paradoxus*) is supported by experimental binding data. We have therefore used more stringent LOD-score thresholds for identifying diverged motifs in *S. cerevisiae*, but this did not eliminate the bias and the difference in the number of predicted genes.

### Quantitative Real-Time PCR

Total RNA was extracted with the MasterPure Yeast RNA purification Kit (EPICENTRE). One microgram of each RNA sample was reverse transcribed with Moloney murine leukemia virus reverse transcriptase (Promega, Madison, WI) and random hexamer primers (Applied Biosystems). Real-time PCR was performed with StepOne real-time PCR machine (Applied Biosystems, Foster City, CA) with Syber Green PCR supermix (Invitrogen). The primers used are described in Table S1.

## Supporting Information

Figure S1 Frequency of interspecific differences in mRNA degradation and mRNA levels as defined by varying thresholds. (a) Percentage of orthologous gene-pairs with differential mRNA levels (blue) and differential mRNA degradation (green) defined by different thresholds of fold-difference. (b) The ratio of percentage of differences in mRNA levels to percentage of differences in mRNA degradation at different thresholds. Dashed lines indicate the same analysis when the frequency of differences in mRNA levels is estimated only among genes without differences (<1.4-fold) in mRNA degradation; this analysis thus estimates the frequency of transcriptional changes divided by the frequency of mRNA degradation changes. At small thresholds (1.1–1.2-fold difference), we find differences at most genes but many of these probably reflect technical variability. At intermediate thresholds (1.4–1.5-fold differences, which are used throughout the article), we find differences at 10%–15% (for mRNA degradation) and ∼50% (for mRNA levels). At higher thresholds (e.g., 2-fold), we find very few differences in mRNA degradation (2%) but many differences in mRNA levels (23%). This analysis suggests a much higher frequency of transcriptional changes, compared with changes in mRNA degradation, and this effect increases with the fold-difference threshold. This effect may be somewhat influenced by the more complex method required for estimation of mRNA degradation, as degradation rates are calculated by the slope of a linear fit to the time course data (after global scaling of each time point), while mRNA levels are estimated directly from a single time-point.
(TIF)

Figure S2 Validation of differential mRNA degradation rates of six genes using quantitative real-time PCR for the two species and for the corresponding hybrid alleles. (a) Measured mRNA levels were normalized by the zero time-point and are shown in blue and red for *S. cerevisiae* and *S. paradoxus* genes, respectively, along with linear least-square fits. (b) Comparison of differential degradation as measured by microarray and quantitative real-time PCR. In all six cases, differential degradation is consistent between the two methods although some quantitative variation is apparent.
(EPS)

Figure S3 Residual transcription cannot account for the observed coupling. The analysis in Figure 2c was repeated for three sets of genes with increasing stringency of the criteria for inclusion of probes: (i) *R* ^2^ thresholds of 0.8, 0.95, and 0.995, and (ii) additional threshold for sum-of-squared residuals of 0.06, 0.03, and 0.015, for set1, set2, and set3, respectively. The resulting three sets contained 2701, 1832, and 615 genes, with set3 having the highest stringency. Notably, the percentage of opposite transcription and mRNA degradation effects increases from set1 to set3, indicating that residual transcription (and other technical effects that cause genes to deviate from exponential decay) does not account for the observed coupling but might in fact cause us to underestimate the effect of the coupling. Note that a similar analysis for genes with considerable deviation from exponential decay (e.g., *R* ^2^ below 0.7) does not reproduce the observed coupling (unpublished data).
(EPS)

Figure S4 Higher expression of Rpb4 in *S. paradoxus* (compared with *S. cerevisiae*) is associated with *trans*-coupled divergence of Rpb4 targets with increased transcription and mRNA degradation in *S. paradoxus*. Expression log_2_-ratios (*S.cer*/*S.par*) are shown for seven subunits of RNA PolII which are included in our analysis, demonstrating a specifically high expression of Rpb4 in *S. paradoxus*. Note that Rpb4 targets are enriched with *trans*-coupled genes for which both transcription and mRNA degradation are higher in *S. paradoxus* (*p*<10^−10^), but are not enriched with *trans*-coupled genes for which transcription and mRNA degradation are higher in *S. cerevisiae* (*p*>0.05), consistent with increased activity of Rpb4 in *S. paradoxus* compared to *S. cerevisiae*.
(TIF)

Figure S5 Coupling is associated with high noise in protein abundance. Cell-to-cell variability in protein abundance (noise), normalized to remove the correlation with average protein abundance (DM values), was taken from Newman et al. [42]. The average and standard error (errorbars) of the normalized noise is shown for all genes, *cis*-coupled and *trans*-coupled genes, TATA-containing and Occupied Proximal Nucleosome (OPN) genes, *cis*-coupled and *trans*-coupled genes after excluding TATA and OPN genes (T/O), and targets of three Ccr4-Not subunits, and Rpb4. These data suggest that (i) coupled genes are associated with high expression noise and that this effect is found both for genes with evolutionary coupling (*cis*-coupled and *trans*-coupled) and for other genes that are regulated by coupling mechanisms (Ccr4-Not and Rpb4/7), (ii) the high noise of coupled genes is comparable to other effects that were previously implicated in increasing expression noise (TATA and OPN), and (iii) the high noise of coupled genes cannot be accounted by enrichment with TATA and/or OPN genes.
(EPS)

Table S1 List of primers (F, forward primer; R, reverse primer).
(DOC)

## Abbreviations

RBP: RNA-binding protein
TF: transcription factor
