# Supplementary figures and images for: Coupled Evolution of Transcription and mRNA Degradation
Source: PLoS Biol. 2011 Jul 19;9(7):e1001106. doi: 10.1371/journal.pbio.1001106 (PMC3139634; doi:10.1371/journal.pbio.1001106)

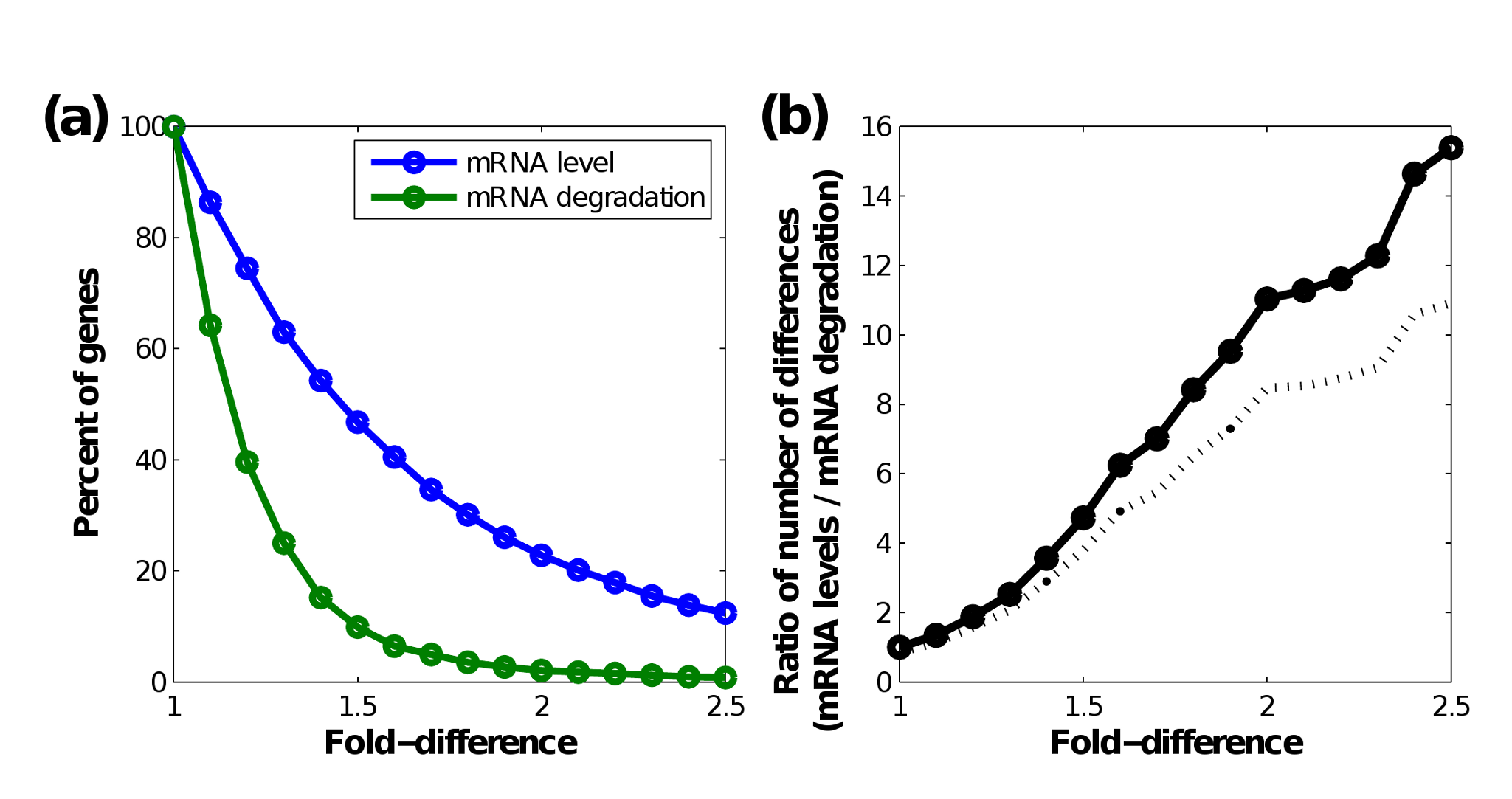

Supplement: Figure S1 — Frequency of interspecific differences in mRNA degradation and mRNA levels as defined by varying thresholds. (a) Percentage of orthologous gene-pairs with differential mRNA levels (blue) and differential mRNA degradation (green) defined by different thresholds of fold-difference. (b) The ratio of percentage of differences in mRNA levels to percentage of differences in mRNA degradation at different thresholds. Dashed lines indicate the same analysis when the frequency of differences in mRNA levels is estimated only among genes without differences (<1.4-fold) in mRNA degradation; this analysis thus estimates the frequency of transcriptional changes divided by the frequency of mRNA degradation changes. At small thresholds (1.1–1.2-fold difference), we find differences at most genes but many of these probably reflect technical variability. At intermediate thresholds (1.4–1.5-fold differences, which are used throughout the article), we find differences at 10%–15% (for mRNA degradation) and ∼50% (for mRNA levels). At higher thresholds (e.g., 2-fold), we find very few differences in mRNA degradation (2%) but many differences in mRNA levels (23%). This analysis suggests a much higher frequency of transcriptional changes, compared with changes in mRNA degradation, and this effect increases with the fold-difference threshold. This effect may be somewhat influenced by the more complex method required for estimation of mRNA degradation, as degradation rates are calculated by the slope of a linear fit to the time course data (after global scaling of each time point), while mRNA levels are estimated directly from a single time-point. (TIF) [file pbio.1001106.s001.tif]

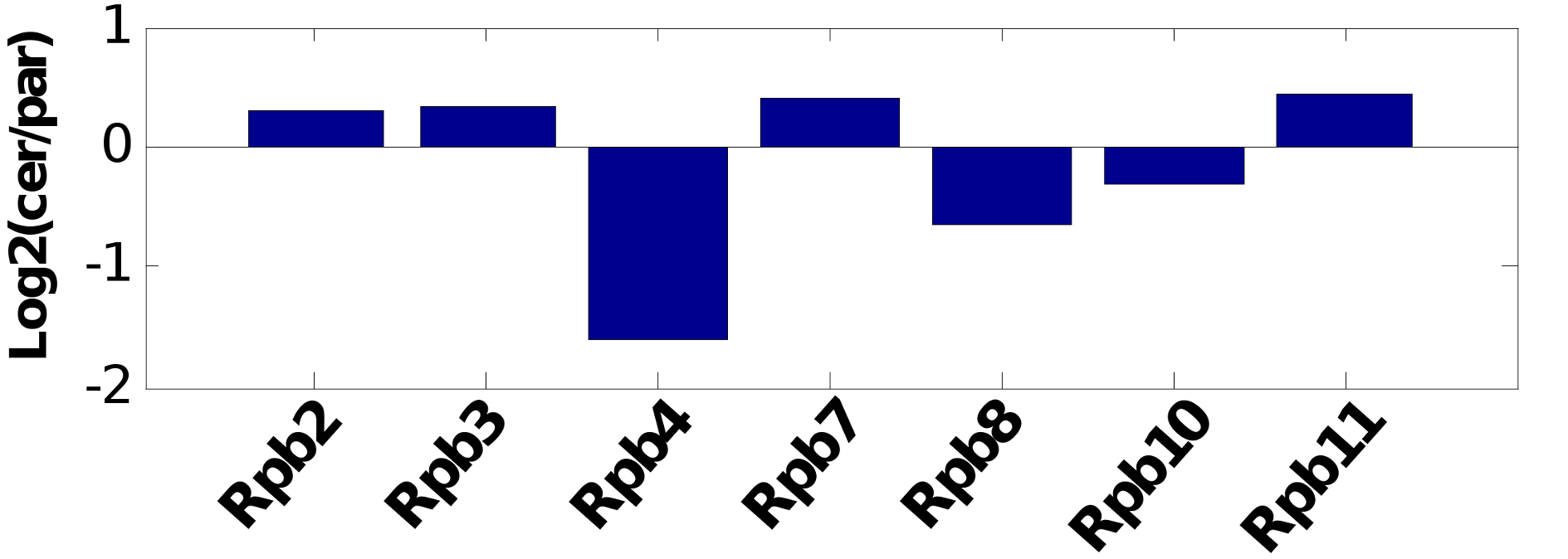

Supplement: Figure S4 — Higher expression of Rpb4 in S. paradoxus (compared with S. cerevisiae) is associated with trans-coupled divergence of Rpb4 targets with increased transcription and mRNA degradation in S. paradoxus. Expression log2-ratios (S.cer/S.par) are shown for seven subunits of RNA PolII which are included in our analysis, demonstrating a specifically high expression of Rpb4 in S. paradoxus. Note that Rpb4 targets are enriched with trans-coupled genes for which both transcription and mRNA degradation are higher in S. paradoxus (p<10−10), but are not enriched with trans-coupled genes for which transcription and mRNA degradation are higher in S. cerevisiae (p>0.05), consistent with increased activity of Rpb4 in S. paradoxus compared to S. cerevisiae. (TIF) [file pbio.1001106.s004.tif]
