# Supplementary material for: Coupled Evolution of Transcription and mRNA Degradation
Source: PLoS Biol. 2011 Jul 19;9(7):e1001106. doi: 10.1371/journal.pbio.1001106 (PMC3139634; doi:10.1371/journal.pbio.1001106)
Supplement: Table S1 — List of primers (F, forward primer; R, reverse primer). (DOC) [file pbio.1001106.s006.doc]

**Supplementary Table 1**.

| ATP7 S.cer F: CGACCTGACAAAGATCAAACCTG |
| --- |
| ATP7 S.cer R: AGGTACGTCCCATTTACCCTTCT |
|  |
| ATP7 S.par F: CGTCGACGATCTGGCAAAG |
| ATP7 S.par R: GTCCCATTTGCCCTTCTTGA |
|  |
| PRP9 S.cer F: AAGTTTATAATGGGCGACGCA |
| PRP9 S.cer R: CCAAGGCATCGCAAGTGATA |
|  |
| PRP9 S.par F: TCTACAATGGACGACGCACC |
| PRP9 S.par R: AATACCCAAGCATCGCAAGTG |
|  |
| GAL83 S.cer F: TCCACAACTGCCACCTCATTT |
| GAL83 S.cer R: GGGCGCCAGACGTATTATCA |
|  |
| GAL83 S.par F: CCTCCACAGCTACCACCACA |
| GAL83 S.par R: GCGCCGGACGTATTATCAGT |
|  |
| Dot6 S.cer F: CCCTCCAGAAAGACCTCGTTG |
| Dot6 S.cer R: TGGGAGTCACATTGGACGG |
|  |
| Dot6 S.par F: CCGTTCGTTCCTCCCACAC |
| Dot6 S.par R: GAGCCCTGTGATGAGGATGGT |
|  |
| ECO1 S.cer F: CAGGGCGGTCGGAATAATAA |
| ECO1 S.cer R: ACCATCCAACGTCCACGACT |
|  |
| ECO1 S.par F: TGACAGGGCGATTGGGATAA |
| ECO1 S.par R: CATCCAACGTCCACGACCA |
|  |
| LAS1 S.cer F: ACGCAACAATCTCAGTTCGCT |
| LAS1 S.cer R: AATCAACAAACCAGGACGGC |
|  |
| LAS1 S.par F: CGCGCTGTCCAAAGAGTACA |
| LAS1 S.par R: TTGCGCCGTAGAGTCCACTA |
